# p38α Mitogen-Activated Protein Kinase—An Emerging Drug Target for the Treatment of Alzheimer’s Disease

**DOI:** 10.3390/molecules29184354

**Published:** 2024-09-13

**Authors:** Jan Detka, Natalia Płachtij, Martyna Strzelec, Aleksandra Manik, Kinga Sałat

**Affiliations:** 1 Department of Pharmacodynamics, Chair of Pharmacodynamics, Jagiellonian University Medical College, 9 Medyczna St., 30-688 Krakow, Poland; jan.detka@uj.edu.pl (J.D.); natalia.plachtij@student.uj.edu.pl (N.P.); aleksandra.manik@student.uj.edu.pl (A.M.); 2 Department of Transplantation, Institute of Pediatrics, Faculty of Medicine, Jagiellonian University Medical College, 265 Wielicka St., 30-663 Krakow, Poland; martyna.strzelec@doctoral.uj.edu.pl

**Keywords:** p38α, mitogen-activated protein kinase (MAPK), Alzheimer’s disease, neuroinflammation, microglia, astrocytes, neurodegeneration, neflamapimod, MW150

## Abstract

Alzheimer’s disease (AD) is a neurodegenerative disorder, characterized by the formation of amyloid β and tau protein aggregates in the brain, neuroinflammation, impaired cholinergic neurotransmission, and oxidative stress, resulting in the gradual loss of neurons and neuronal function, which leads to cognitive and memory deficits in AD patients. Chronic neuroinflammation plays a particularly important role in the progression of AD since the excessive release of proinflammatory cytokines from glial cells (microglia and astrocytes) induces neuronal damage, which subsequently causes microglial activation, thus facilitating further neurodegenerative changes. Mitogen-activated protein kinase (MAPK) p38α is one of the key enzymes involved in the control of innate immune response. The increased activation of the p38α MAPK pathway, observed in AD, has been for a long time associated not only with the maintenance of excessive inflammatory process but is also linked with pathophysiological hallmarks of this disease, and therefore is currently considered an attractive drug target for novel AD therapeutics. This review aims to summarize the current state of knowledge about the involvement of p38α MAPK in different aspects of AD pathophysiology and also provides insight into the possible therapeutic effects of novel p38α MAPK inhibitors, which are currently studied as potential drug candidates for AD treatment.

## 1. Introduction

Alzheimer’s disease (AD) is a neurodegenerative disorder and one of the most common causes of dementia. As estimated, there are around 50 million AD patients worldwide nowadays, and this number will double every 5 years, reaching 152 million by the year 2050. AD has a negative impact not only on individuals but also on their families and has serious economic consequences with estimated global costs of USD 1 trillion annually [1,2,3].

As a multifactorial disease and one of the most prevalent forms of cognitive decline, AD is characterized by a gradual decrease in neuronal function and neuronal loss, which causes a significant impairment of behavioral and cognitive functions including memory, attention, and reasoning [1,2,4]. In AD, two types of neuropathological changes can be distinguished, namely positive lesions, appearing due to an accumulation of neurofibrillary tangles (NFTs) formed by hyperphosphorylated tau protein, amyloid β (Aβ) plaques, dystrophic neurites, neuropil threads, and negative lesions, which occur due to brain tissue atrophy, i.e., neural, neuropil, and synaptic loss. Several factors underlie these pathologies observed in the course of AD, including inter alia neuroinflammation, oxidative stress (OS), and the loss of cholinergic neurons [1,5,6,7,8].

Neuroinflammation is an inflammatory response of the brain and spinal cord that can be caused by various pathological insults, such as infection, trauma, ischemia, and toxins. On the one hand, this process is regarded as a protective inherent host mechanism involved in the protection of the brain and restoration of its function against various infections and injuries, but, on the other hand, this phenomenon involves all cells of the central nervous system (CNS), including glial cells and neurons, to release potentially toxic compounds, which cause neurotoxicity, synaptic dysfunction, neuronal death, and the inhibition of neurogenesis. Microglia and astrocytes are further activated in response to CNS cell damage and continue to release proinflammatory mediators, including proinflammatory cytokines: interleukin-1β (IL-1β), interleukin- 6 (IL-6), tumor necrosis factor α (TNF-α), various chemokines (CXCL-1, CCL2, CCL5), nitric oxide (NO), and prostaglandins. In addition to this, capillary endothelial cells and infiltrating blood cells are strongly involved in neuroinflammation, in particular, when the blood–brain barrier (BBB) is damaged [6,7]. According to the Common Alzheimer’s Disease Research Ontology (CADRO), neuroinflammation is one of the key phenomena underlying AD pathology and, therefore, it is regarded as a relevant target for novel drug candidates for AD [9].

The expression of inflammation-related genes and the release of inflammatory mediators is regulated by various intercellular pathways involving protein kinases, among which, mitogen-activated protein kinase (MAPK) family member p38α (p38α MAPK) appears to play a particularly important role in the progression of the innate immune response to stress conditions. This protein kinase is one of the key contributors to glial cell-mediated neuroinflammation, including microglia and astrocytes. p38α MAPK stimulates the nuclear factor kappa-light-chain-enhancer of activated B cells (NF-κB) and increases glutamate excitotoxicity along with synaptic plasticity disruption. It is activated by several proinflammatory mediators, such as cytokines, chemokines, and bacterial lipopolysaccharide (LPS). Moreover, studies also show that p38 MAPK is involved in tau protein phosphorylation [7], and the activation of this kinase cascade has been shown to be an important mediator for the generation of Aβ and its neurotoxicity [10].

The involvement of p38 signaling in the development of AD has been extensively reviewed by many authors over the years [3,11]. This review will summarize recent scientific advances concerning the potential role of p38α MAPK in the progression of pathophysiological changes characteristic of the development of AD. In particular, we will focus on the role of p38α MAPK as a drug target for novel anti-AD drug candidates, and we will summarize currently available data on the pharmacological properties, clinical efficacy, and potential harms of disease-modifying agents acting with p38α MAPK inhibition.

## 2. Overview of Mitogen-Activated Protein Kinase Signaling

The regulation of protein activity through their phosphorylation by protein kinases is one of the major mechanisms of signal transduction in all living cells. This allows them to process multiple external signals and produce a coordinated response to the received stimuli. To date, a wide variety of protein kinases has been discovered, with over 500 different types identified in human cells. Among all of them, mitogen-activated protein kinases (MAPKs) are the most abundant and thus the most extensively studied kinase group. These enzymes were proven to exert pivotal roles in the control of vital cellular activities, including the regulation of gene expression, protein biosynthesis, cell cycle, motility, metabolism, differentiation, immunity, and survival [12].

MAPKs are a family of proline-directed, serine-threonine protein kinases (EC 2.7.11.24), highly conserved throughout the evolutionary tree and found in all eukaryotic organisms, from yeast to humans. MAPK activation is a three-tier process with upstream involvement of other groups of serine-threonine protein kinases, namely MAPK kinase kinases (MAPKKKs or MAP3Ks) and MAPK kinases (MAPKKs or MAP2Ks). In response to extracellular stimulus, initially, MAP3Ks are activated either by phosphorylation by other kinases downstream from extracellular receptors, or by interactions with small Ras/Rho GTPases. Activated MAP3Ks phosphorylate MAP2Ks, which in turn activate MAPK via dual phosphorylation of threonine and tyrosine residues within a highly conserved Thr-Xaa-Tyr (where Xaa states for any amino acid) structural motif of the activation loop [13,14]. More than one MAP3K is capable of activating selected MAP2K, and, consequently, each MAPK can be phosphorylated by multiple MAP2Ks. Such a three-tier arrangement of MAPK activation cascade enables the amplification and modulation of the extracellular signals and their appropriate integration, which allows the cells to generate well-coordinated responses to multiple, often divergent stimuli [15,16].

In mammals, 14 different MAPKs have been characterized. These proteins can be divided into seven distinct subgroups, among which, four types are regulated via a conventional three-tier activation mechanism and dual phosphorylation. These include extracellular-signal-regulated kinases 1 and 2 (ERK1/2), ERK5, c-Jun N-terminal kinases 1, 2, and 3 (JNK1/2/3), and p38 isoforms α, β, γ and δ [16]. ERK1/2 kinases are activated primarily in response to growth factors, hormones, and other mitogens but also, to some extent, by cytokines and stress conditions. Instead, JNK1/2/3 and p38 isoforms are also referred to as ‘stress-activated protein kinases’, since, in large part, they are triggered by a plethora of environmental stressors, DNA damage, cytokines, and other proinflammatory factors and regulate immune and stress responses, cell differentiation, and apoptosis [16,17]. Physiological outcomes resulting from the action of a particular MAPK pathway may be variable and are strongly dependent on external and internal conditions and cell type [18].

The dysregulation of MAPK signaling pathways is a hallmark of many pathological conditions. In particular, because of its key role in the regulation of innate immune response, the chronic activation of the p38 MAPK pathway is a common feature in various inflammatory and autoimmune diseases and cancer [19]. Numerous experiments conducted over the years directly link ongoing chronic inflammatory processes with the development and/or progression of multiple sclerosis [20], epilepsy [21], mental illnesses like depression [22], bipolar disorder [23], and schizophrenia [24] as well as neurodegenerative diseases, such as amyotrophic lateral sclerosis [25], Parkinson’s disease (PD) [26], and various forms of dementia, including AD [27].

## 3. Characteristics of p38α MAPK Signaling Cascade

Kinase p38α, also known as mitogen-activated protein kinase 14 (MAPK14), was originally named p38, and it was first identified as a protein phosphorylated in response to LPS stimulation and osmotic stress in mammalian cells [28]. In mammalian genomes, p38α kinase is encoded by the *MAPK14* gene. It is the most abundant isoform, expressed in a variety of tissues, while the expression of other isoforms, namely β, γ, and δ, is somewhat more restricted to the particular tissue type [29]. The family of p38 MAP kinases is activated by cellular stressors, e.g., inflammatory cytokines, UV irradiation, osmotic pressure, and oxidative stress [28,30,31,32,33,34,35], and p38 MAP kinases downstream targets are composed of cytoskeletal and scaffold proteins, transcription factors, molecular chaperones, metabolic enzymes, and signaling factors [35,36,37,38,39].

**Figure 1:**
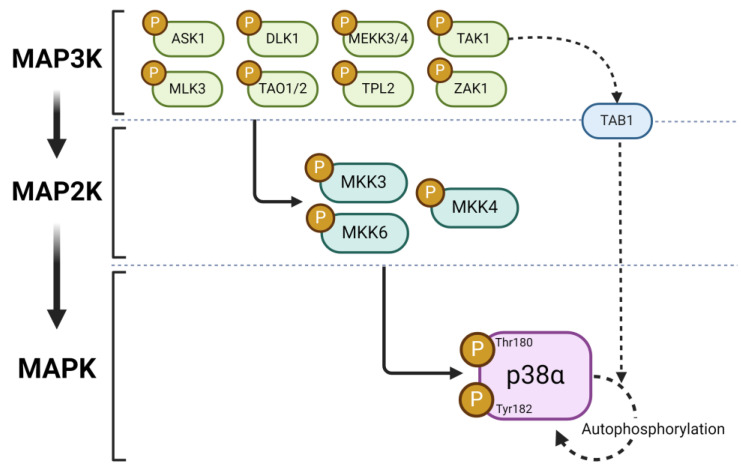
Schematic diagram depicting main routes for activation of p38α mitogen-activated protein kinase (p38α MAPK) in the central nervous system. The canonical, three-tier route of activation with the involvement of MAPK kinase kinases (MAP3K) and MAPK kinases is marked with a solid line, while the alternative pathway involving transforming growth factor β-activated kinase 1 (TAK1) and transforming growth factor β-activated protein kinase binding protein 1 (TAB1) to induce p38α MAPK autophosphorylation is marked with a dotted line. Abbreviations: ASK1—apoptosis signal-regulating kinase 1, DLK1—dual-leucine-zipper-bearing kinase 1, TAO1/2—thousand-and-one amino acid kinase 1 and 2, TPL2 tumor progression loci 2, MLK3—mixed-lineage kinase 3, MEKK3/4—MAPK/ERK kinase Kad 3 and 4, ZAK1—leucine zipper and sterile-α motif kinase 1. Created with BioRender.com (accessed on 7 August 2024).

The activation of p38 occurs through the dual phosphorylation of Thr-180 and Tyr-182 residues located on the Thr-Gly-Tyr structural motif of the activation loop within kinase subdomain VIII. The phosphorylation of these two residues leads to conformational changes, which relieve steric blocking and stabilize the activation loop in a more open position and thus facilitate substrate binding [40]. In mammals, MKK3 and MKK6 are the two main MAP2Ks responsible for p38 activation. MKK6 is specific towards all p38 isoforms, whereas MKK3 can activate only p38α, p38γ, and p38δ, but not p38β. Additionally, p38α can also be phosphorylated by MKK4, which is normally involved in the activation of JNK [40,41,42]. The activation of MKK6 and MKK3 occurs, in turn, with the participation of several different MAP3Ks, the most important of which are apoptosis signal-regulating kinase 1 (ASK1), dual-leucine-zipper-bearing kinase 1 (DLK1), transforming growth factor β-activated kinase 1 (TAK1), thousand-and-one amino acid protein kinases 1 and 2 (TAO1/2), tumor progression loci 2 (TPL2), mixed-lineage kinase-3 (MLK3), MAPK/ERK kinase Xia 3 and 4 (MEKK3 and 4), and leucine zipper and sterile-α motif kinase 1 (ZAK1) [29,43]. There are also alternative, non-canonical pathways for p38 activation. The first one involves the direct interaction of p38 with transforming growth factor-β-activated protein kinase 1-binding protein 1 (TAB1), which leads to kinase autophosphorylation at Thr-180/Tyr182, and it can be further stimulated by another adapter protein TNF receptor-associated factor-6 (TRAF6) [44] (Figure 1). Another alternative activation pathway occurs exclusively in T cells, where Tyr-323 is phosphorylated by TCR-proximal tyrosine kinases ZAP70 (ζ -chain associated protein kinase of 70 kDa) and p56lck, allowing for p38 autophosphorylation within the activation loop [45].

## 4. Physiological Functions of p38α MAPK Signaling in Central Nervous System

In the CNS, the kinase p38α isoform is the most abundant and thus most extensively studied out of the whole kinase p38 family. p38α MAPK is expressed in neurons [46], and its function is strictly conditioned by the location—neurite (axon) or soma [47]. In neurons, this kinase pathway can be triggered by, among other factors, *N*-methyl-d-aspartate (NMDA) and metabotropic glutamate receptors (mGluRs). It regulates a wide array of cellular processes and functions, including long-term potentiation (LTP) and Long Term Depression (LTD) [48], ion channel activity [49], synaptic adhesion [50], neuronal cytoskeleton modulation [51], axonal transport [52], neurotoxicity, and survival [53,54], as well as neuronal differentiation [55] with the underlined fact that the neuronal differentiation is a mechanism in which potentially p38 kinases could be mutually compensated [55,56].

p38α kinase is also expressed by astrocytes [57,58]. During brain injury or neurotoxic conditions, p38α kinase regulates astrocytic neuroinflammatory responses by promoting cytokine and chemokine production [58,59,60], as well as NF-κB signaling and production of reactive oxygen species (ROS) [61]. In light of some recent work, it might be speculated that astrocytic p38α may also be involved in the modulation of astrocyte-to-neuron crosstalk and influence neuronal activity. In a study by Navarrete et al. [62], the activation of the p38α MAPK pathway in astrocytes promoted NMDA-dependent LTD at the CA3-CA1 synapses of mice, and this effect was later abolished only after knock-out of astrocytic but not neuronal p38α MAPK.

p38α kinase is considered to be a key p38 isoform in microglial activation. An animal model of the traumatic brain injury deletion of p38α in microglia led to the attenuation of the inflammatory reaction, reduction in cytokine levels, and a decline in microglial recruitment to the damaged sites of brain regions [63].

In oligodendrocytes—myelin-producing cells of the CNS—p38α is associated with a multistage process of developing a lipid-rich insulating layer that wraps axons, providing their support and the rapid propagation of action potential. The inhibition of p38α kinase inhibition in oligodendrocytes restricts their differentiation [64] and developmental myelination [65]. Interestingly, p38α kinase comes out as an inhibitor of remyelination in a cuprizone-induced demyelination model [66,67].

**Figure 2:**
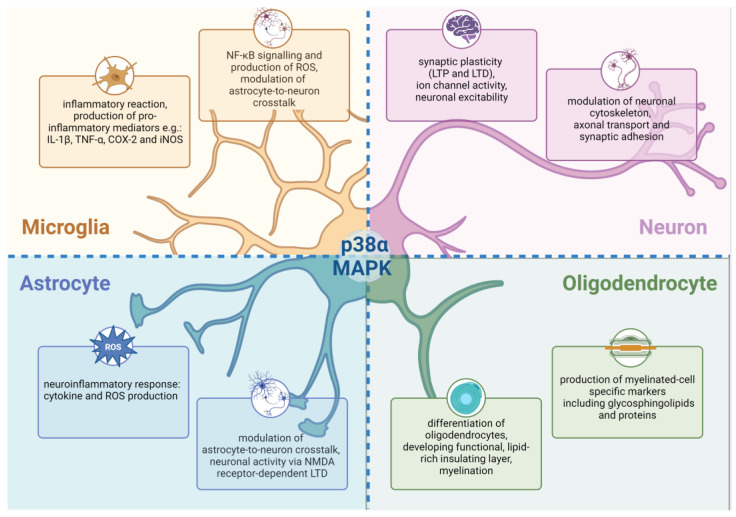
Physiological role of p38α MAPK in the central nervous system (CNS) and its functions in different types of CNS cells. Abbreviations: COX-2—cyclooxygenase-2; iNOS—inducible nitric oxide synthase; IL-1β—interleukin-1β; LTD—long-term depression; LTP—long-term potentiation; NF-κB—nuclear factor kappa-light-chain-enhancer of activated B cells; NMDA—*N*-methyl-d-aspartate; ROS—reactive oxygen species; TNF-α—tumor necrosis factor α. Created with BioRender.com (accessed on 7 August 2024).

A brief summary of the key physiological functions of p38α MAPK in the CNS is presented below (Figure 2).

## 5. Involvement of p38α MAPK in the Progression of Neuroinflammatory Process in AD

p38α MAPK can act as an important mediator of the inflammatory process since its first established role was the regulation of biosynthesis of IL-1 and TNF-α in monocytes, which are major proinflammatory cytokines [31]. However, it is important to note that the duration of p38α-mediated pathway activation is critical for the final physiological effect it would cause in the cell. In physiological conditions, the p38 MAPK activation process is usually quick and transient, but the question arises of what may happen in the conditions of chronic activation [13], especially considering the fact that p38 MAPK pathways were originally described as activated by stress [68]. Most recently, it has been shown that the abnormal accumulation of the phosphorylated form of p38α is a pathological sign, and higher levels of phosphorylated p38α have been linked to downstream signal transduction and can contribute to chronic inflammation [69]. As p38 MAPKs are one of the main regulatory mechanisms involved in upregulating cytokine production, they can also take part in the dysregulation of inflammatory responses and cause disturbances in CNS homeostasis. This leads to neurotoxicity and neuroinflammation [70], which is a common contributing factor for a variety of neurodegenerative disorders. Therefore, p38α MAPK is intensely researched as a potential novel therapeutic target for alleviating the neuroinflammatory component of numerous CNS diseases.

p38α MAPK is abundantly expressed in the CNS, compared to other kinases included in this family, as it can be found in all neural cell types: neurons, astrocytes, and microglia [56], the last of which are the main, innate immune cells responding to inflammation in the brain [71]. They do so by changing their phenotype, which causes a shift in their functions [72]—in normal conditions, they exhibit a phagocytic function in removing damaged neurons, but in the activated state they show a secretory role [73]. Cellular receptors present on microglial cells, e.g., toll-like receptors (TLRs) can recognize specific molecular patterns associated with either damage or pathogens, thus inducing microglial activation and production of mediators of inflammation such as IL-1β, IL-6, TNF-α, and nitric oxide [74], which are also commonly known activators of the p38α MAPK pathway together with LPS, UV light, heat shock, and other stress stimuli [68]. It is then clear that p38α creates some kind of a linkage between microglial cellular responses and inflammatory stimuli [75]. It has been proven by multiple studies—Bachstatter et al. showed the connection between p38α and the regulation of IL-1β and TNF-α overproduction [69], Xing et al. reported that p38α MAPK in microglia plays a critical role in neurotoxicity mediated by these cells [76], and Chen et al. showed the inhibition of p38α suppressed the activation of NLR family pyrin domain containing the 3 (NLRP3) inflammasome pathway, which takes part in the innate immune response and neurodegeneration [77]. What is interesting is that p38α can also influence microglial autophagy, thus also influencing the production of pro-inflammatory cytokines—He et al. demonstrated that p38α inhibits autophagy of microglia via interactions with unc-51 like autophagy-activating kinase 1 (ULK1) [78].

Astrocytes, as another type of glial cells, can also contribute to the inflammation in the CNS via the p38α MAPK pathway. Their primary roles in the CNS focus on keeping the homeostasis of ions and neurotransmitters, protecting the nervous tissue against ROS, and constituting the formation of the BBB [79]. During inflammation, astrocytes can interact with microglia, oligodendrocytes, neurons, and endothelial cells in the BBB, but they can respond to injury and inflammation by themselves as well, indicating that signaling pathways, including the p38α MAPK pathway, in these cells, might play a role in the CNS inflammation [80]. Indeed, experiments by Lo et al. presented a role played by p38α in the expression of genes encoding specific cytokines, chemokines, and adhesion molecules in astrocytes [58], while Revuelta et al. showed that pharmacological inhibition of p38α in aged astrocytes, where the levels of this kinase are naturally higher, mitigates the symptoms characteristic of neurodegenerative disorders [81].

**Figure 3:**
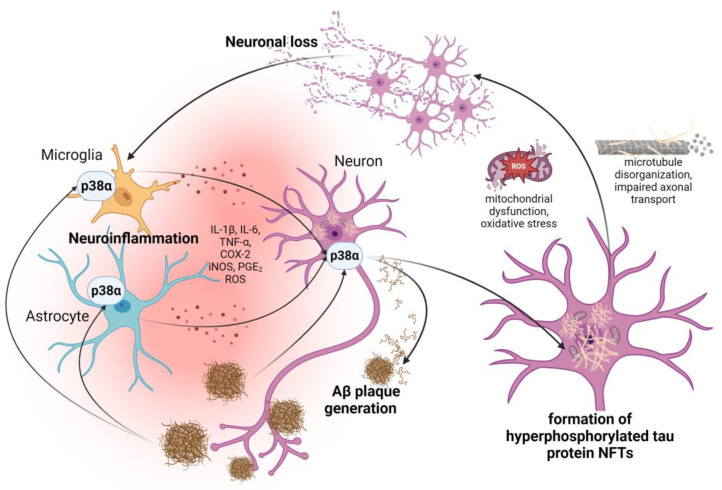
Involvement of p38α MAPK pathway in the development of main pathophysiological features of Alzheimer’s disease. Abbreviations: Aβ—amyloid β; COX-2—cyclooxygenase-2; iNOS—inducible nitric oxide synthase; IL-1β—interleukin-1β; IL-6—interleukin-6; NFTs—neurofibrillary tangles; PGE_2_—prostaglandin E_2_; ROS—reactive oxygen species; TNF-α—tumor necrosis factor α. Created with BioRender.com (accessed on 7 August 2024).

The p38α MAPK pathway has also been linked to many important neuronal functions, as it may modulate neuronal excitability and synaptic plasticity. However, under chronic inflammatory conditions, it is also reported that elevated p38α activation in neurons, i.e., due to the activation of residual microglia and astrocytes and cytokine release, may lead to the increase in the formation of proteinopathies characteristic of AD, such as Aβ plaques and hyperphosphorylated tau protein, NFTs. This, in turn, may lead to neural cell damage, axonal and synaptic dysfunction, and neuronal cell death [7,82] (Figure 3).

## 6. The Role of p38α MAPK Pathway in the Generation and Deposition of Aβ Plaques

One of the most characteristic features of AD is Aβ plaques composed of Aβ_1–40_ and Aβ_1–42_ forms, which are generated after proteolysis of amyloid precursor protein (APP) by membrane-bound enzymes—secretases: α-secretase, β-secretase (β-site APP-cleaving enzyme (BACE1)) [83], and γ-secretase, which is a complex composed of four proteins: presenilin-1 (PSEN1), nicastrin, anterior pharynx-defective 1 (APH-1), and presenilin enhancer 2 (PEN2) [84]. The formation and accumulation of Aβ plaques induce the activation of both microglia and astrocytes, which stimulates them to release proinflammatory cytokines in order to remove the abnormal proteins. This prolonged cytokines release causes neuroinflammation resulting in cell death [85] and synaptic dysfunctions [86]. It has been shown by Hensley et al. [87] that the active phosphorylated form of p38 kinase is localized in plaques and NFTs within both neurons and microglia in AD brains, but not in the healthy donors’ brains, suggesting that the p38 MAPK pathway is upregulated in the course of AD. A study by Schnöder et al. showed that partial deletion of the p38α MAPK reduces Aβ generation by BACE1 degradation [83], while Colié et al. reported that the downregulation of p38α in AD in vivo model improves memory, reduces Aβ deposits, and modulates astrogliosis, microglia activation, and neurogenesis [10].

The receptor for advanced glycation end products (RAGE) is one of the major mediators of both the neurotoxic and neuroinflammatory effects of Aβ. This receptor belongs to an immunoglobulin superfamily, binds different types of Aβ oligomers, and is responsible for their elimination from the brain, across the BBB. RAGEs are present in both microglia and astrocytes, as well as in neurons, and the activation of RAGEs by Aβ induces a cascade, leading to the activation of p38α MAPK in all these cells. This activation leads to the activation of microglia and astrocytes, accompanied by the release of proinflammatory cytokines and other inflammatory mediators. In neurons, RAGE-dependent p38α activation (occurring directly or indirectly by proinflammatory cytokines) results in an increase in tau phosphorylation and metabolic and mitochondrial dysfunction, leading to the impairment of axonal transport, LTP generation, and cell death [56,88]. The RAGE-dependent signaling pathway was also found to regulate the activity of β- and the γ-secretase cleavage of APP to generate Aβ, with the engagement of p38 MAPK and glycogen synthase kinase-3β (GSK-3β) [89].

## 7. p38α MAPK in the Phosphorylation of Tau Protein and the Formation of Neurofibrillary Tangles

NFTs built by hyperphosphorylated tau protein filaments are another major characteristic pathological structure observed in AD. In normal conditions, tau protein promotes the assembly and stability of microtubules, but in the course of AD, the abnormal protein aggregation causes microtubule dysfunction resulting in the impaired functioning of neurons, i.e., affecting protein transport across axons and proper organization of synapses [90]. Tau oligomers are also known to cause disturbances in energy metabolism, ATP production, and mitochondrial function, affect protein degradation by lysosomes, and have harmful effects on the cell genome by acting on protein-DNA complexes [91].

p38α is also suggested to be one of over 20 different serine-threonine protein kinases involved in tau phosphorylation [92,93]. Pharmacological inhibition of p38 reverses cytotoxic effects induced by extracellular tau protein on microglia and enhances tau phagocytosis performed by these cells [94]. Treatment with MW181—a small-molecule inhibitor of p38α MAPK—decreased tau phosphorylation in an hTau transgenic mouse model of AD, significantly increased the expression of synaptic protein synaptophysin, and improved working memory in rodents. It also decreased MAPK-activated protein kinase 2 (pMK2) and phosphorylated activating transcription factor 2 (pATF2), which are downstream substrates of p38α, and it reduced levels of proinflammatory cytokines IL-1β and interferon-γ (IFN-γ) [95].

## 8. Involvement of p38α MAPK in Oxidative Stress-Induced Damage in AD

Cellular damage caused by ROS is another important mechanism involved in the pathophysiology of AD. All cells have a defensive system based on mitochondrial enzymes such as superoxide dismutase (SOD), glutathione peroxidase (GPx), and catalase (CAT), as well as antioxidants such as glutathione, which protect the cells from the damage caused by circulating free radicals. The disproportion between ROS accumulation and their effective removal is known by OS [96]. The brain is one of the most vulnerable organs and one of the easiest targets for ROS, of which increased levels are especially detectable in aged brains. OS-induced mutations in the mitochondrial DNA and impaired glucose metabolism lead to the dysfunction of synaptic transmission and ultimately to neuronal death. The number of damages increases with age, which promotes aging and also propagates neurodegeneration [97,98]. Lipid peroxidation associated with Aβ peptides is considered the most significant type of oxidative damage. The brain consists mostly of lipids, more precisely cholesterol, which is why it is particularly vulnerable to peroxidation. The most important indicator of the lipid peroxidation process is 4-hydroxy-2-trans-nonenal (HNE), associated with proteins. As a highly active alkenal, it changes the conformation of proteins present in the brain and reduces their activity. This significantly contributes to disease progression and modifications of both Aβ and tau proteins [99]. Increased production of ROS, as well as the decreased ability of their removal, occur mostly due to impairment in mitochondrial function in AD patients [100]. OS leads to neuronal damage and cell death via ROS accumulation, and the occurrence of this phenomenon can also lead to the activation of the p38α MAPK pathway, as there is evidence that mitochondrial dysfunction resulting in OS is one of the earliest signs of neuronal loss in the brain during AD progression [101]. ROS can target several elements of the p38α MAPK pathway, including the ASK1 protein, which is one of the major upstream MAP3K activators of this kinase cascade [102]. The activation of p38α MAPK results in the increased production of proinflammatory cytokines, which are the main cause of neuroinflammation present in the AD brain [103] and in tau protein phosphorylation leading to, as explained before, formation of NFTs—the hallmark of AD. It has also been researched that Aβ deposition and OS are linked, although it is not clear yet which one comes first—on the one hand, Aβ induces OS by interfering with mitochondrial activity and the NF-kB pathway, but on the other, OS increases the production of Aβ and might actually be an early event in the transition from normal aging to AD pathology [104].

## 9. Impact of p38 MAPK on Cholinergic Neurotransmission in the Brain

In addition to generalized negative effects on axonal transport and synaptic function, increased p38 MAPK activity is known to directly interfere with the cholinergic system in brain regions crucial for memory and cognitive processing, such as the frontal cortex and hippocampus. Cholinergic neuronal loss and signaling dysfunctions in rat brains were associated with increased p38 MAPK activation in microglia and astrocytes with the increased release of IL-1β and elevated expression of cyclooxygenase-2 and inducible nitric oxide synthase (iNOS) in microglia and astrocytes [105]. More recently, it has been shown that Aβ can be internalized by cholinergic neurons via interactions with α7 nicotinic acetylcholine receptor (α7nAChR), known to play a key role in cognitive processing. The internalized Aβ-α7nAChR is deposited in mitochondria and lysosomes and, in turn, activates the p38 MAPK cascade and is associated with an increase in apoptotic marker levels in the mouse brain [106].

## 10. Available Treatment for AD

At present, there are three classes of drugs officially approved to treat AD. These comprise cholinesterase inhibitors (donepezil, rivastigmine, and galantamine), antagonists of NMDA receptors (memantine), and anti-Aβ monoclonal antibodies (aducanumab, lecanemab, and, approved in July 2024, donanemab) [107].

Cholinesterase inhibitors are further classified as reversible, irreversible, and pseudo-reversible inhibitors. They act by blocking the enzymes (acetylcholinesterase and butyrylcholinesterase), which physiologically hydrolyze acetylcholine, and this results in increased levels of this neurotransmitter in the synaptic cleft. Since overactivation of NMDA receptors promotes cell death and synaptic dysfunction, the NMDA receptor antagonist, memantine, restores the normal activity of this receptor. This was also found to be beneficial in the treatment of AD. It is worth noting that the therapeutic effects of these two anti-AD drug classes are only symptomatic, and these medications improve cognitive functions and decrease selected symptoms of AD without modifying the disease itself, and importantly, in contrast with anti-Aβ monoclonal antibodies, none of these drugs is able to cure or prevent the disease. Cholinesterase inhibitors are mainly used to treat mild to moderate symptoms of AD, while memantine (alone or in combination with cholinesterase inhibitors) is used for the treatment of moderate to severe AD [1,5,6,7,108].

## 11. Drug Candidates for AD—The Past and the Future

Considering the low therapeutic efficacy of cholinesterase inhibitors and memantine, novel treatment options for this neurodegenerative disorder are being evaluated. The research is focused on the assessment of disease-modifying drug candidates, mainly compounds targeting various proteins implicated in AD pathology.

### 11.1. Disease-Modifying Therapeutics

The rationale for using disease-modifying therapeutics (DMTs) to attenuate and slow down the progression of AD is to modify several pathophysiological mechanisms implicated in the development of AD. Several experimental DMTs have been designed, and these compounds entered the clinical trials. 

For example, AN-1792 is regarded as the first-in-class active immunotherapy strategy for AD. It consists of the synthetic full-length Aβ peptide combined with QS-21 immune adjuvant. In line with preclinical data showing that immunization with Aβ_1–42_ peptide can prevent or reverse the development of the neuropathological hallmarks of AD (amyloid plaque formation, neuritic dystrophy, neuronal loss, gliosis, and impaired performance of experimental animals in behavioral assays), it was hypothesized that AN-1792 would induce an immune response that would remove brain amyloid deposition. AN-1792 entered phase 2a clinical trials in patients with mild to moderate AD, but it was discontinued in 2002 due to brain inflammation resulting in aseptic meningoencephalitis, which appeared in 6% of the patients [1].

Other experimental therapies were also developed but, similarly to AN-1792, they failed in the clinical trials. For example, the anti-Aβ antibodies (solanezumab and bapineuzumab), α-secretase modulators and activators that stimulate the cleavage of APP, γ-secretase inhibitors (semagacestat, avagacestat, tarenflurbil), β-secretase inhibitors (lanabecestat, verubecestat, atabecestat, umibecestat, also known as CNP520) were unsuccessfully assessed, and the failure of such potential novel therapies was attributed to several issues, including inefficacy, starting therapy too late, and inappropriate drug dose regimens used [1].

Human anti-Aβ monoclonal antibodies that bind with high affinity to aggregated Aβ (aducanumab, lecanemab, donanemab, gantenerumab, and crenezumab) are also DMTs, of which aducanumab, lecanemab, and donanemab have been registered as anti-AD drugs [108,109,110,111,112].

Also, chaperones (heat shock proteins: Hsp60, Hsp70, Hsp90) became a subject of interest as potential drug targets and DMT strategies to treat AD by blocking the aggregation process of misfolded proteins, like amyloidogenic proteins (i.e., Aβ and tau), and promoting their degradation [113].

Tau aggregation inhibitors have been also investigated as promising DMTs. For instance, inhibitors of GSK3β (tideglusib) reduce tau hyperphosphorylation and block tau deposition [114].

Also, saracatinib (AZD0530), a protein kinase inhibitor that acts by inhibiting the brain Src/abl family of kinases, has demonstrated good efficacy in improving memory in transgenic mice and is currently evaluated in a phase 2 clinical trial [115].

#### 11.1.1. DMTs—Focus on Neuroinflammation

As mentioned above, neuroinflammation is one of the key factors underlying the development of AD [5,6,7]. In view of this, anti-inflammatory agents (non-steroidal anti-inflammatory drugs, peroxisome proliferator-activated receptor-γ activators, minocycline, and anti-TNFα drugs: thalidomide, etanercept) were evaluated for their potential utility as DMTs in AD, but the results obtained were discouraging [5,6,7,116,117,118].

This low efficacy of the tested experimental therapies has become a starting point for further exploring inflammation-related molecular targets for new drugs for AD. Below, we will focus on compounds that inhibit p38α MAPK.

##### Neflamapimod

**Figure 4:**
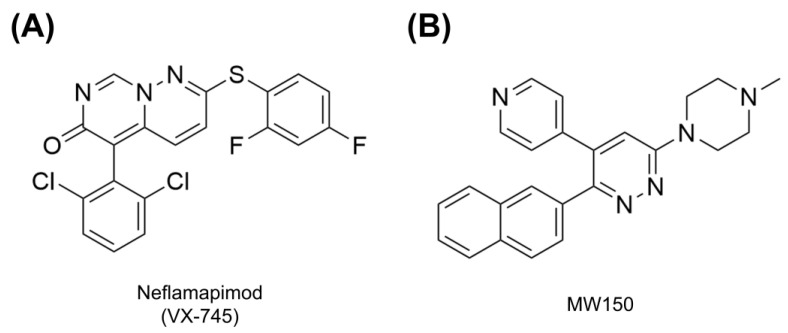
Chemical structures of two p38α MAPK inhibitors currently undergoing phase 2 clinical trials in AD: (**A**) neflamapimod (VX-745) and (**B**) MW150. Created with ChemDraw (Version 23.1.1; Revvity Signals Software, Waltham, MA, USA).

Neflamapimod (also known as VX-745, Figure 4A) was initially developed as a modulator of inflammatory diseases, including rheumatoid arthritis, but later on, it was repurposed for development as a potential drug candidate for AD. It is an orally available small molecule selective inhibitor of p38α MAPK—the enzyme that is highly expressed in microglia and other brain cells. This drug candidate reaches higher concentrations in the CNS than in peripheral blood.

In neurons, the expression of p38α MAPK is low in healthy subjects, but its expression is increased under cellular stress conditions and disease states [119,120]. The activation of p38α MAPK induces the release of proinflammatory cytokines in response to a variety of stressors, including Aβ42, and is involved in neuroinflammation. Hence, it was assumed that neflamapimod might attenuate the progress of AD.

In animal models, it has been demonstrated that neflamapimod alters microglial activation from a pro-inflammatory to a phagocytic state and improves mitochondrial function and synaptic transmission, which also leads to improved memory. In rodents, neflamapimod was able to lower IL-1β concentrations and improved the spatial learning assessed in the Morris water maze task [118,121]. In an ischemic stroke model in rats, a 6-week treatment with neflamapimod starting at 48 h after reperfusion accelerated the recovery of sensory and motor function, and it increased brain-derived neurotrophic factor concentration in the brain [122]. Neflamapimod stimulated blood vessel dilatation and reduced vascular inflammation [123].

Neflamapimod attenuated cholinergic dysfunction by reducing Rab5 activation and prevented the degeneration of basal forebrain cholinergic neurons, which are the main source of acetylcholine in the brain. In vivo, it improved performance in novel object recognition and open-field tasks [7,118,119,121,124].

Two open-label phase 2 trials assessing neflamapimod started in 2015. The first one compared its two doses: 40 mg or 125 mg, given orally for 6 weeks to patients with mild forms of AD. The second one enrolled patients with an AD diagnosis who were administered neflamapimod, either 40 mg or 125 mg, twice daily for 12 weeks. Both trials aimed to assess its effect on the brain amyloid plaque load and episodic memory function. In general, neflamapimod was safe, and no serious adverse effects were reported. It showed some positive effects on both amyloid removal and episodic memory in AD patients [118,121,125].

Another phase 2 trial started in 2017, and it enrolled 161 patients with mild AD. This proof-of-concept trial compared a six-month therapy with 40 mg neflamapimod taken twice daily to a placebo. The results of this study announced in 2019 were discouraging as its primary endpoint (improvement of episodic memory) was not met. The analysis of the results obtained for neflamapimod showed that it was safe but not more efficacious than a placebo. However, neflamapimod lowered the biomarkers of synaptic dysfunction in the cerebrospinal fluid. Taken together, these results indicated that a longer study of neflamapimod used at a higher dose level is needed to assess its effects on AD progression [126].

Further, phase 2 studies assessing the efficacy of neflamapimod in patients suffering from dementia with Lewy bodies, AD, and Huntington’s disease were run in the years 2018–2021 [119]. Some of them have already been completed, but the results have not been made public, yet. However, it has to be also noted that in 2019, the FDA granted a fast-track designation to neflamapimod for dementia with Lewy bodies. A study by Jiang and colleagues also showed that neflamapimod treatment demonstrated some improvement in functional mobility and a dementia rating scale for basal forebrain cholinergic degeneration, and this agent was well-tolerated with no drug-associated treatment discontinuation [119].

In May 2023, another phase 2 clinical trial assessing neflamapimod in dementia with Lewy bodies began. The aim of this study is to compare the efficacy of a 16-week treatment with 40 mg neflamapimod given three times daily to a placebo in 160 participants. The study is now running at 29 sites in the U.S. and The Netherlands, through August 2024 [127].

##### MW150

MW150 (also known as MW01-18-150SRM; 6-(4-methylpiperazin-1-yl)-3-naphthalen-2-yl-4-pyridin-4-ylpyridazine, Figure 4B) is the second p38α MAPK inhibitor currently developed in phase 2 clinical trial for the treatment of AD. MW150 is a CNS-penetrant and orally available compound [128].

In the APP/PS1 mouse model of amyloidosis, treatment with MW150 prevented the development of memory deficits without affecting amyloid plaque accumulation. In APP/PS knock-in mice, the administration of MW150 to older mice suppressed memory deficits [128,129]. MW150 was also able to inhibit the release of proinflammatory cytokines from glia in mouse models, and it selectively modulated neuroinflammatory responses associated with pathology progression in microglia without inducing the pan-suppression of normal physiological functions of microglia [119,130]. It also improved cognitive functions in mice [120] measured using passive avoidance and novel object recognition tasks (authors’ unpublished data).

In a mouse model of autism spectrum disorder, MW150 normalized social behavior and physiological disturbances resulting from hyperserotonemia [131].

In 2018, a phase 1 study assessing the activity of MW150 started. It proved that MW150 was safe, well-tolerated, and reached promising blood levels after oral administration to healthy volunteers.

In January 2022, a phase 2 study with MW150 began. It tested the efficacy of this compound in patients with mild to moderate AD. The trial is expected to be completed in August 2024 [132].

Apart from neflamapimod and MW150, numerous other compounds showed the ability to inhibit p38α MAPK, e.g., ginsenoside Rg1, trolox, SB239063, macranthoin, pinocembrin, linalool, astaxanthin. Their biological activities and potential therapeutic applications are described elsewhere (e.g., [3,133,134,135,136]).

#### 11.1.2. p38α MAPK Inhibitors—Safety Concerns

Preclinical studies and clinical trials evaluated the ability of neflamapimod and MW150 to improve memory and reduce neuroinflammation. At the same time, the possible risks and harmful effects resulting from p38α MAPK inhibition were preliminarily assessed. The issues raised could be regarded as potential limitations of these innovative p38α MAPK-based therapies.

Some of these hypothetical toxic effects might be of particular concern in the elderly (i.e., patients with AD, in particular, those with AD accompanied by comorbidities or patients exposed to polypharmacy, i.e., patients at high risk of drug–drug interactions). For example, Singh and colleagues [137] used cell-based in vitro assays to study the potential toxicity of p38α MAPK inhibitors (BIRB796, VX-745, and SB203580). This study showed the possible hepatotoxicity of these compounds. This effect was due to the inhibition of p38 kinase, which elevated the level of phospho c-Jun N-Terminal Kinase (pJNK) in LPS-stimulated human cells. The activation of pJNK has been linked to increased caspase-3 activation, which contributes to liver toxicity and tumor growth. In addition to this, the p38 MAPK inhibitors tested in LPS-induced HepG2 cells increased the levels of hepatic aminotransferase as compared to LPS used alone. Taken together, the authors concluded that hepatotoxicity should be regarded as a potential adverse effect of p38α MAPK inhibitors.

Safety issues were also investigated by Bengal and colleagues [137], who pointed out that p38 MAPK pathways modulated the metabolic adaptation of skeletal muscle to exercise, and these enzymes were involved in various aspects of whole-body energy metabolism. This resulted from increasing glucose transport into the tissue, elevating glycolytic and citric acid cycle flux, and raising the number of mitochondria. p38 MAPK signaling mediates these positive effects by phosphorylating the transcription factors and coactivators involved in the metabolism of carbohydrates. In view of this, the inhibition of p38 MAPK might negatively influence these phenomena. Moreover, the inhibition of p38 MAPK might block hepatic gluconeogenesis and reduce hepatic glucose release.

Also, a study by Dambach [138] focused on the assessment of the potential adverse effects of p38 MAPK inhibitors. Since p38 mediates developmental, differentiation, and proliferation processes, it plays an important role in a variety of cellular processes. Hence, it was suggested that p38 MAPK inhibitors might demonstrate some adverse effects on cell proliferation and differentiation, and these harmful activities were likely to result from the enzyme inhibition. This undesirable effect might be of key importance during fetal or neonatal development, but it should also be taken into consideration in the adult population undergoing repair or adaptive processes. 

In clinical trials (NCT02423122 and NCT02423200), the safety and tolerability of neflamapimod were assessed in patients with a mild form of AD. In both studies, no serious safety issues were identified [139]. However, one has to note that, previously, some unacceptable CNS-related toxicity was demonstrated for neflamapimod used at high doses in animal studies [139].

The potential adverse effects of neflamapimod were also analyzed by Prins and colleagues [126], who reported that neflamapimod was well tolerated in clinical trials, and some of the observed adverse effects, such as, for example, hypokalemia and plasma cell myeloma, were considered unrelated to treatment. Other p38α MAPK inhibitors, such as MW150, showed good safety and tolerability in phase 1 clinical trials (NCT04120233, NCT02942771) [140], but these studies suggested some risk for elevated liver enzymes. For p38 MAPK inhibitors tested in the rheumatoid arthritis trials, a transient increase in liver enzymes was also seen in 10–15% of patients, and this was considered to be a drug-class effect [141]. It is also noteworthy that numerous inhibitors of p38 MAPK that were considered drug candidates for the treatment of rheumatoid arthritis, chronic obstructive pulmonary disease, and asthma also failed to pass clinical trials due to limited efficacy and potentially harmful adverse effects due to their unspecific, off-target action [142]. Target specificity appears to be an especially important safety issue in the context of AD treatment since individual p38 isoforms are known to exert slightly different biological effects in the CNS [56].

## 12. Conclusions

Although the primary cause of AD has not yet been elucidated, there is now no doubt that chronic neuroinflammation can significantly contribute to its progression. This phenomenon was shown to facilitate the development of both positive lesions, such as Aβ and tau proteinopathies, as well as negative lesions like neurodegeneration associated with AD. Currently applied therapeutic strategies for AD aimed at intensifying cholinergic transmission or neuronal activity show limited clinical efficacy. Over the years, many alternative therapeutic strategies for AD treatment have been considered, and novel drug candidates targeting misfolded proteins have also proven to be ineffective in most cases. Therefore, increasing attention is being focused on inhibiting inflammation-related pathways as a disease-modifying therapeutic strategy for AD. 

The p38α MAPK signaling pathway is one of the key regulatory mechanisms implicated in neuroinflammation in the course of AD. Studies show that this signaling cascade can either trigger or be triggered by the release of cytokines and other proinflammatory mediators and is activated in all types of CNS cells during the disease course. Extensive studies on the p38α MAPK role in the pathogenesis of AD clearly associate its increased activation with both the formation of positive lesions such as Aβ plaques and hyperphosphorylated tau protein NFTs, as well as with negative lesions, such as impairment in neuronal and synaptic functions and OS-induced neuronal damage. Moreover, in many preclinical studies, blocking the p38α MAPK pathway, either by genetic knockdown or by selective small-molecule inhibitors, has been continuously shown to alleviate neurodegenerative brain changes and also improve memory and cognitive function in experimental animals. Two of the selective p38α MAPK inhibitors, neflamapimod (VX-745) and MW150, are currently under phase 2 clinical trials. 

Studies with neflamapimod that began in 2015 presented some therapeutic efficacy of this compound in alleviating the severity of the cognitive symptoms of AD. A positive effect related to the decreased levels of biological markers of AD was also demonstrated for this agent. Also, a phase 2 clinical trial for the second drug candidate, namely MW150, began in 2018, and its results are yet to be released.

In view of the key role of Aβ in the early stage of AD pathophysiology and considering the involvement of p38α MAPK signaling in the formation of Aβ, it seems that a p38α MAPK-targeted treatment might offer the best therapeutic (i.e., disease-modifying) effects if introduced during the early stage of AD development. Such an approach would influence the progression of the disease and slow down the appearance of its main symptoms. Of note, recently, neflamapimod was assessed in a 24-week double-blind, placebo-controlled clinical trial (NCT03402659) in 161 patients with early-stage AD, and this study demonstrated its effectiveness in reducing selected markers of AD [122,126].

Taken together, the increased activity of the p38α MAPK pathway in the brain can be a major contributor to the development of AD, and, therefore, this enzyme should be considered a promising therapeutic target for specific small-molecule inhibitors—drug candidates for AD. Since the activation of this protein kinase is associated not only with neuroinflammation but also with many other pathophysiological features of AD, a novel therapeutic approach based on the selective inhibition of p38α MAPK could potentially modify multiple mechanisms leading to neurodegeneration and thus be more effective in alleviating the symptoms of cognitive decline and memory loss in patients suffering from AD.

## Data Availability

No new data were created or analyzed in this study.

## Abbreviations

AD	Alzheimer’s disease
ASK-1	apoptosis signal-regulating kinase 1
α7 nAChR	α7 nicotinic acetylcholine receptor
Aβ	amyloid β
BACE1	β-site APP-cleaving enzyme (β-secretase)
BBB	Blood–brain barrier
CCL2	C-C motif chemokine ligand 2
CCL5	C-C motif chemokine ligand 5
CNS	central nervous system
CXCL-1	C-X-C motif chemokine ligand 1
DMTs	disease-modifying therapeutics
GSK-3β	glycogen synthase kinase-3β
IL-1β	interleukin-1β
IL-6	interleukin-6
iNOS	inducible nitric oxide synthase
LPS	Lipopolysaccharide
LTD	long-term depression
LTP	long-term potentiation
MAP2K	MAPK kinase
MAP3K	MAPK kinase anchor
MAPK	Mitogen-activated protein kinase
MKK3	MAPK kinase 3
MKK4	MAPK kinase 4
MKK6	MAPK kinase 6
NFTs	neurofibrillary tangles
NF-κB	nuclear factor kappa-light-chain-enhancer of activated B cells
NLRP3	NLR family pyrin domain containing 3 inflammasome
NMDA	*N*-methyl-D-aspartate
NO	nitric oxide
OS	oxidative stress
PGE_2_	prostaglandin E_2_
pJNK	phospho c-Jun N-Terminal Kinase
RAGE	receptor for advanced glycation end product
ROS	reactive oxygen species
TLR	Toll-like receptor
TNF-α	tumor necrosis factor α
